# Abnormalities in the zinc-metalloprotease-BDNF axis may contribute to megalencephaly and cortical hyperconnectivity in young autism spectrum disorder patients

**DOI:** 10.1186/s13041-014-0064-z

**Published:** 2014-09-03

**Authors:** Jae-Young Koh, Joon Seo Lim, Hyae-Ran Byun, Min-Heui Yoo

**Affiliations:** 1 Neural Injury Research Lab, Asan Institute for Life Science, University of Ulsan College of Medicine, Seoul, Korea; 2 Department of Neurology, University of Ulsan College of Medicine, 388-1 Poongnap-Dong Songpa-Gu, Seoul 138-736, Korea

**Keywords:** Autism spectrum disorder (ASD), Zinc, Metalloprotease, Brain-derived neurotrophic factor (BDNF)

## Abstract

Whereas aberrant brain connectivity is likely the core pathology of autism-spectrum disorder (ASD), studies do not agree as to whether hypo- or hyper-connectivity is the main underlying problem. Recent functional imaging studies have shown that, in most young ASD patients, cerebral cortical regions appear hyperconnected, and cortical thickness/brain size is increased. Collectively, these findings indicate that developing ASD brains may exist in an altered neurotrophic milieu. Consistently, some ASD patients, as well as some animal models of ASD, show increased levels of brain-derived neurotrophic factor (BDNF). However, how BDNF is upregulated in ASD is unknown. To address this question, we propose the novel hypothesis that a putative zinc-metalloprotease-BDNF (ZMB) axis in the forebrain plays a pivotal role in the development of hyperconnectivity and megalencephaly in ASD.

We have previously demonstrated that extracellular zinc at micromolar concentrations can rapidly increase BDNF levels and phosphorylate the receptor tyrosine kinase TrkB via the activation of metalloproteases. The role of metalloproteases in ASD is still uncertain, but in fragile X syndrome, a monogenic disease with an autistic phenotype, the levels of MMP are increased. Early exposure to lipopolysaccharides (LPS) and other MMP activators such as organic mercurials also have been implicated in ASD pathogenesis. The resultant increases in BDNF levels at synapses, especially those involved in the zinc-containing, associative glutamatergic system may produce abnormal brain circuit development. Various genetic mutations that lead to ASD are also known to affect BDNF signaling: some down-regulate, and others up-regulate it. We hypothesize that, although both up- and down-regulation of BDNF may induce autism symptoms, only BDNF up-regulation is associated with the hyperconnectivity and large brain size observed in most young idiopathic ASD patients.

To test this hypothesis, we propose to examine the ZMB axis in animal models of ASD. Synaptic zinc can be examined by fluorescence zinc staining. MMP activation can be measured by in situ zymography and Western blot analysis. Finally, regional levels of BDNF can be measured. Validating this hypothesis may shed light on the central pathogenic mechanism of ASD and aid in the identification of useful biomarkers and the development of preventive/therapeutic strategies.

## Introduction

Autism spectrum disorder (ASD) is a heterogeneous developmental disorder of the brain characterized by impaired communication, abnormal sociability, restricted interests, and stereotyped behaviors [1]. In most ASD cases, autistic symptoms manifest by 3 years of age [2], indicating that the disease process starts during early childhood. ASD is strongly associated with male sex, with boys being affected about 4 to 5-times more frequently than girls [3]. One notable clinical feature is the apparent recent increase in the prevalence of ASD; whereas the frequency of ASD was considered to be as low as 5 in 10,000 in 1980s [4], the most recent data from the Center for Disease Control and Prevention report it to be as high as 1 in 68 (CDC 2014). Whether this increase is real or instead reflects heightened awareness of ASD is uncertain. Unfortunately, current treatments for ASD are limited and generally provide only symptomatic relief.

Although the majority of ASD cases remain idiopathic, recent years have witnessed dazzling progress towards an understanding of the underlying neurobiological mechanisms. In about 10% of cases, the etiology of ASD has a monogenic basis, such as fragile X syndrome (FMR1 gene), Rett syndrome (MECP2 gene) or tuberous sclerosis (TSC1 and 2 genes), each of which causes autistic symptoms in affected individuals [5]-[7]. However, even in idiopathic cases, ASD appears to be under a strong genetic influence. For instance, monozygotic twins have a concordance rate of 58–60%, whereas for dizygotic twins the corresponding rate is 21–27% [8]. Genetic studies conducted to date have identified more than 500 genes that may be associated with ASD [9]. Among the better known examples are PTEN (phosphatase and tensin homolog); SHANK (SH3 and multiple ankyrin repeat domains) 1, 2 and 3; NLGN (neuroligin) 1 and 3; and MEF (myocyte enhancer factor) 2A and 2D [10]-[12]. These ASD candidate genes, together with FMR1, TSC1 (tuberous sclerosis 1) and MECP2 (methyl CpG binding protein 2) are likely involved in neurogenesis, synaptic signaling, and synaptic plasticity.

ASD is considered primarily a disorder of brain circuitry. Hence, many investigators have sought to explain ASD symptoms by searching for abnormal synaptic functions. For instance, Geschwind and colleagues suggested that ASD brains have an imbalance in excitatory/inhibitory neurotransmission [13]. In addition, postmortem studies have reported abnormal cellular organization in the cortex of ASD patients [14],[15]. Also, since neuroligins, neurexins and Shank family proteins are involved in synapse formation and synaptic transmission, defects in the genes encoding these proteins are likely to result in reduced synaptic activity in certain brain regions. Consistent with this supposition, earlier studies using functional magnetic resonance imaging (fMRI) demonstrated that ASD brains may have decreased connectivity compared with typical brains [16]. A corollary of this hypoconnectivity theory is that enhancement of synaptic activity may be therapeutic for ASD patients [17]. However, arguing against this idea, a number of subsequent studies have reported quite the opposite: young ASD brains may instead be hyperconnected, either regionally or globally [18],[19]. The existing literature suggests that hypoconnectivity may be a late phenomenon [20],[21]; thus, increasing synaptic transmission may not be an appropriate therapeutic approach, at least in young ASD patients. Thus, in this article, we attempt to frame the mechanism involved in initiating ASD in the context of a new hypothesis that can explain large brain sizes and hyperconnectivity based on findings reported in the literature and our own preliminary results.

### Features of ASD that suggest increased neurotrophism

#### Hyperconnectivity in young ASD brains

As discussed above, earlier studies on brain connectivity in ASD suggested hypoconnectivity as the core functional pathology of ASD. Using fMRI, Just et al. first provided experimental evidence for hypoconnectivity in ASD [16],[21]. These researchers proposed that a diminished degree of information integration and synchronization might form the neurobiological foundation of ASD. Subsequent fMRI studies reported supportive findings [22],[23]. Hence, because of its conceptual appeal and the availability of some supporting evidence, this theory was initially hailed as the “first firm finding” on ASD pathobiology [24]. However, as additional results accumulated, this early consensus faded and divergent ideas have emerged. Given seemingly incompatible results, Muller and colleagues critically analyzed 32 published studies and concluded that fMRI results may vary depending on methodological differences such as task-dependence and filter choice [25]. Moreover, because most such studies examined an adult population, it remained unclear whether brain connectivity status in ASD is heterogeneous from the beginning or changes with aging.

More recently, Supekar and colleagues examined young ASD patients using task-free fMRI and found hyperconnectivity throughout the brain [18]. In addition, Keown and colleagues, using resting-state functional connectivity MRI, found hyperconnectivity in posterior brain regions of young ASD patients [26]. Both groups found a correlation between hyperconnectivity and symptom severity, indicating that the hyperconnectivity is not only a general characteristic of young ASD patients but is also likely responsible for ASD symptoms. These studies strongly support the theory that brain hyperconnectivity is the main underlying functional abnormality, at least in young ASD patients.

Consistent with the hyperconnectivity theory, Markram and Markram proposed the “intense world hypothesis”, initially based on their findings obtained in the valproate model of autism in rats [27]. They found that newborn rats exposed to valproate during fetal development exhibited enhanced fear processing and memories [28]. These changes were attributed to enhanced neuronal activity and plasticity in brain areas such as the amygdala and neocortex. Hence, they suggested that autistic brains are easily trapped in a “painfully intense world”.

Taken together, these studies strongly suggest that, at least during early brain development, overall hyperconnectivity rather than hypoconnectivity is the main circuit abnormality in most ASD brains.

#### Large brains

Kanner first noted that some autistic children have macrocephaly [29]. Since then, other investigators have found that macrocephaly is statistically over-represented in ASD compared with the general population [30]. Interestingly, it was reported that, whereas the head circumferences of ASD patients are not larger at birth, they grow more rapidly between 6 and 14 months of age [31]. MRI studies have confirmed that large heads correlate with large brains, although the increases may not be uniform across brain areas [32]-[34]. Postmortem studies have also shown that the brains of ASD patients possess an excess number of neurons, especially in the prefrontal cortex [35]. This finding is consistent with the observation that frontal lobes are enlarged more than occipital lobes in ASD patients [36]. Volumetric studies indicate that both white and gray matter are enlarged [36]-[39].

Some cross-sectional studies indicate that the increased brain growth in ASD may be age dependent. Courchesne et al. reported that increased gray and white matter volume may occur largely during early childhood, followed by normalization to control values at older age [31]. This age dependence appears consistent with the above-mentioned putative changes in brain connectivity—that is, hyper connectivity in young patients and hypoconnectivity in older patients.

Although it is not certain whether more neurons and/or glial cells (astrocytes, microglia, oligodendrocytes) are generated during the brain growth spurt in ASD, various trophic factors, including epidermal growth factor (EGF) and insulin-like growth factor 1 (IGF1), have been proposed to be involved [40],[41]. However, evidence for a role for EGF or IGF1 in ASD is mixed. Although some studies have reported increased levels of EGF in ASD [40], others have reported the opposite [42], leaving the molecular basis for the brain overgrowth in young ASD patients undetermined. Recently, however, investigators have noted that BDNF levels are often increased in various models of ASD and may underlie the brain overgrowth.

#### BDNF upregulation

BDNF is a potent neurotrophic factor that belongs to the neurotrophin (NT) family. Other members of the family include nerve growth factor (NGF), NT3, and NT4/5. The mature form of BDNF is generated from proBDNF by proteolysis [43],[44]. Whereas proBDNF preferentially acts at the NGF receptor, p75NTR, mature BDNF tends to selectively activate TrkB. A number of cell culture studies have demonstrated that activation of TrkB induces increased neuronal hypertrophy and survival [45]. Consistent with this, injection of BDNF into the developing neocortex induces abnormally increased neurogenesis [46]. In addition, BDNF/TrkB signaling contributes to synaptic development and plasticity, neurite outgrowth and dendritic spine formation, which are possible anatomical correlates of hyperconnectivity in ASD.

In 2001, Nelson et al. first reported that the levels of certain trophic factors, including BDNF, are increased in the blood of young ASD patients [47]. Additional interest in BDNF in ASD was prompted by the observation that the gene involved in Rett syndrome, a neurodevelopmental disorder exhibiting autistic features, encodes MECP2 [48], which regulates BDNF expression in the brain [49]. Mutations in the *MECP2* gene result in a reduction in BDNF levels in the brain, indicating that a deficiency in BDNF may contribute to the pathology of Rett syndrome [50]. Though seemingly opposite to expectations, this result nevertheless attracted attention to the possible role of BDNF in ASD. Subsequently, a number of studies reported varying results regarding BDNF levels in the serum or brains of ASD patients. One possible explanation for this variation is that BDNF levels may be dependent on age: whereas BDNF production is enhanced during neonatal periods [47],[51],[52], it may be reduced in adult patients [53].

Although it is difficult to pinpoint the underlying bases for the widely variable findings regarding BDNF in ASD, the above-mentioned age dependence may play a role. Animal models of ASD also exhibit variable changes in BDNF levels. In a mouse model of fragile X syndrome (*Fmr1*-knockout mice), BDNF levels are decreased in the cortex but increased in the hippocampus [54]. However, TrkB receptor expression as well as calcium increases are increased in these mice following BDNF exposure (ibid), indicating that BDNF-TrkB signaling is upregulated in Fmr1-deficient cells. Other ASD models in which BDNF upregulation has been demonstrated include the valproate model [55] and PTEN model [56]. Mutations in PTEN likely increase BDNF levels by decreasing BDNF clearance. Notably, the transcription factors MEF2A and 2D, mutations of which are implicated in ASD, act at the promoter region of the BDNF gene; knockdown of Mef2d results in the upregulation of BDNF [57]. In general, models that show increased BDNF levels are correlated with human counterparts that exhibit increased brain connectivity and size.

In stark contrast, *Mecp2*-null mice, a model for Rett syndrome, clearly show reduced BDNF expression. Also, mice lacking the cation-proton antiporter NHE6 exhibit attenuated TrkB signaling [58]; notably, mutations in NHE6 cause Christianson syndrome, which exhibits autistic features. Hence, it appears that genetic mutations that cause ASD may be classified into two categories: one with increased BDNF levels and hyperconnectivity (e.g., FMR1, PTEN, MEF2A), and one with decreased BDNF levels and hypoconnectivity (MECP2 and NHE6). Defects in MECP2 or NHE6, which result in decreased BDNF expression or TrkB signaling in *Mecp2*- and *NHE6*-null mice, respectively, appear to be associated with microcephaly in the corresponding human disorders, Rett syndrome and Christianson syndrome [59],[60]. This apparent correlation between BDNF levels and connectivity seems to support the hypothesis that upregulation of BDNF signaling during a critical period of brain development may contribute to the large brain size and hyperconnectivity noted in young idiopathic ASD patients. The key question then becomes: how is BDNF upregulation triggered?

### Formulation of the hypothesis

#### Are metalloproteases involved?

A variety of stimuli influence BDNF expression/signaling [61]. Synaptic activity, which causes depolarization and calcium influx, is a known stimulus for BDNF upregulation [62]. Brain injuries, such as seizures, ischemia and inflammation, also enhance BDNF expression, likely through similar mechanisms [63]-[66]. Cleavage of proBDNF into mature BDNF can be mediated by furin in the endoplasmic reticulum [67]; in the extracellular milieu, BDNF processing takes place via plasmin [68]. Mizoguchi et al. recently showed that MMPs may be involved in the conversion of pro-BDNF to BDNF, thus enhancing TrkB signaling [69]. Because BDNF can upregulate MMP levels [70], a positive feedback loop may exist between metalloproteases and BDNF.

Despite a potential link between metalloproteases and BDNF, the possibility that metalloproteases are involved in the pathogenesis of ASD has not yet been systematically addressed. However, there is some circumstantial evidence to support such a link. In fragile X syndrome (FXS), dysfunction of the gene product FMRP leads to MMP9 over-activation [71]. The observation that minocycline, an MMP inhibitory antibiotic, has some therapeutic efficacy [72] suggests that MMP9 over-activation plays a causal role in FXS. Hence, MMP9 might possibly contribute to the BDNF upregulation and megalencephaly that are associated with FXS [73]. Consistent with this, FMRP dysfunction leads to an increase in the number of synaptic boutons and dendritic spines in animals and humans [74],[75], an alteration that is ameliorated by minocycline [76]. In addition, MMP9 levels in idiopathic ASD cases were found to be elevated compared with controls [77]. Notably, plasma levels of secreted amyloid precursor protein-α (sAPPα), a product of the α-secretase membrane metalloprotease ADAM10 [78],[79], are increased in severe ASD patients [80]. Hence, the activity of various metalloproteases may be increased in ASD.

**Figure 1:**
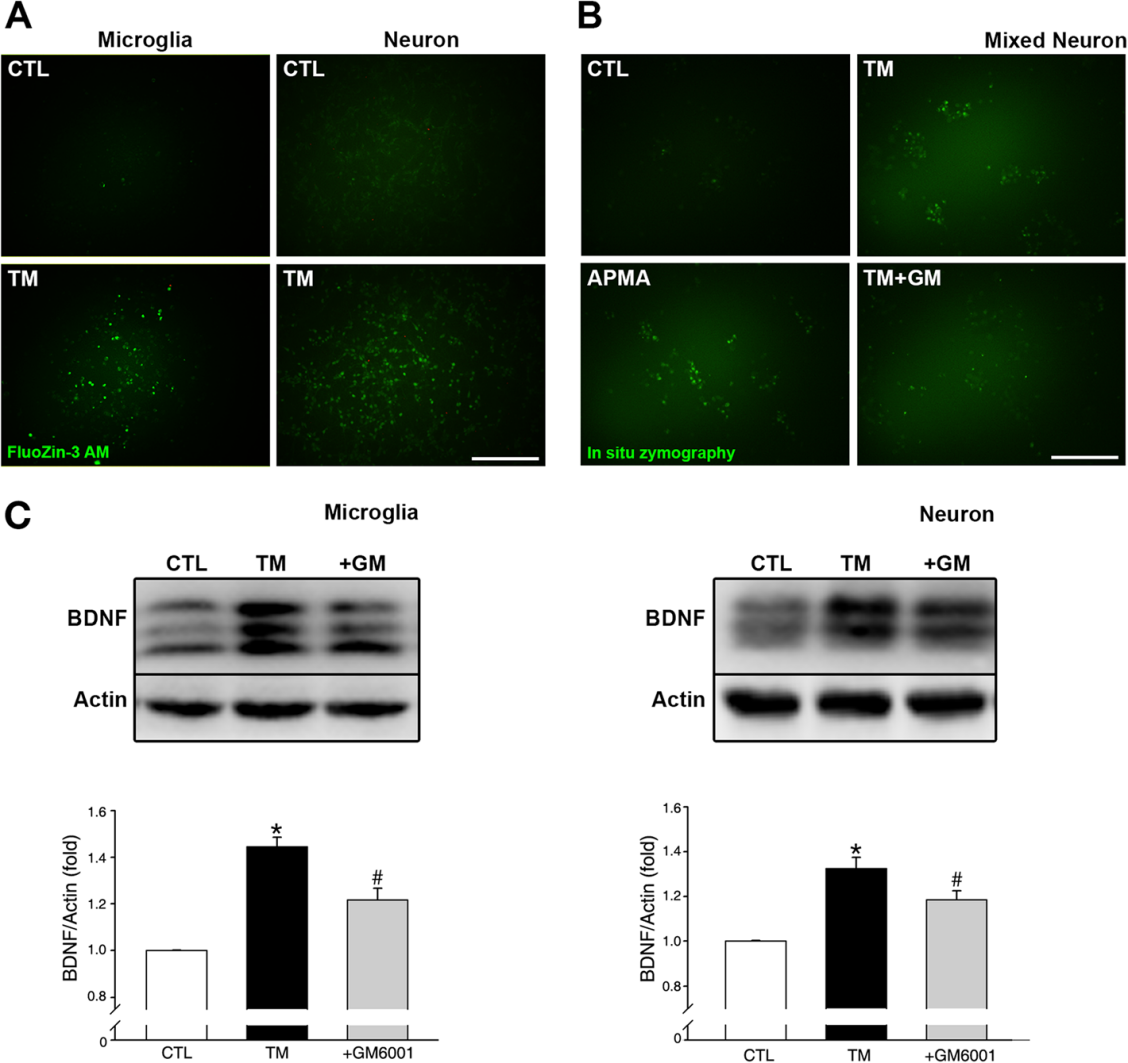
**Thimerosal increases BDNF levels in cultured microglia and neurons: Mediation by zinc and metalloproteases. (A)** Primary cultures of microglia and neuron were exposed to thimerosal (TM; 1 μM) for 60 min, and the resulting increase in free zinc was analyzed by FluoZin-3 AM staining. Scale bar: 100 μm. Representative of n = 4 individual experiments. **(B)** Mixed cortical cell cultures were exposed to thimerosal (TM; 1 μM) for 60 min. In situ zymography for MMP showed that TM exposure induced MMP activation in neuronal cells. APMA, a potent MMP activator, also induced similar increases in MMP activity, whereas addition of GM6001 (GM), an MMP inhibitor, reduced increases in MMP activity by TM. Scale bar: 100 μm. The image is representative of n = 3 individual experiments. **(C)** Western blots of microglial (top left) and neuronal (top right) cells with anti-BDNF antibody revealed that treatment with TM increased levels of BDNF in both cell types. Addition of GM6001 (GM) attenuated the increase in BDNF. The image is representative of n = 4 individual experiments. Bottom row graphs represent respective densitometric analysis for each blots, in which BDNF/Actin for CTL (control) was set as 1. *p < 0.05 vs. CTL, #*p* < 0.05 vs. TM.

Organic mercury compounds, especially thimerosal (included historically in vaccines as a preservative), has been implicated as a potential culprit in the pathogenesis of ASD (Bernard et al., [81]). The unifying theme is that these compounds may induce neurotoxicity in the developing brain through diverse mechanisms [82]. Whereas a number of subsequent studies would appear to have disproved this connection [83]-[86], some recent studies continue to suggest that thimerosal may be a risk factor for ASD [87]-[90]. Consistent with these reports, repeated injections of thimerosal in young rats was shown to produce an autistic phenotype in a strain-dependent manner [91]. These observations motivated us to explore a possible connection between thimerosal and metalloproteases. Our preliminary data obtained from cortical cell cultures indicate that thimerosal activates MMP and increases the levels of free zinc, MMP, and BDNF in microglia and neurons (Figure 1). Hence, it may be worthwhile testing the effects of thimerosal on MMPs and BDNF in animal brains. Notably, other environmental contributors to ASD, such as LPS and testosterone, may also be capable of activating metalloproteases in the brain [92],[93].

#### Synaptic zinc, metalloproteases, and ASD

If increased metalloprotease activity contributes to ASD pathogenesis, it is logical to ask what might trigger it. Among the known environmental risk factors that might be invoked as possible metalloprotease activators are brain inflammation (LPS), thimerosal (organic mercurials), and testosterone. To this list of factors we would propose adding zinc, more specifically, synaptic zinc at zinc-containing glutamatergic (gluzinergic) synapses [94], because of its role in metalloprotease activation and increased BDNF signaling in ASD.

A substantial amount of free or labile zinc is present in synaptic vesicles of certain glutamatergic neurons, mainly in terminals of cortical and hippocampal associative fibers; glutamatergic projection neurons do not appear to have synaptic zinc [94]. Zinc transporter 3 (ZnT3) plays an essential role in zinc accumulation in synaptic vesicles, as evidenced by the fact that genetically deleting the ZnT3 gene results in complete disappearance of synaptic vesicle zinc [95],[96]. Upon neuronal activation, synaptic zinc is released along with glutamate [97] and likely serves diverse signaling functions. For instance, zinc modulates the activity of ion channels, such as NMDA (N-methyl-D-aspartate) and GABA (γ-aminobutyric acid) receptors [98],[99], and contributes to synaptic plasticity, playing a role in long-term potentiation (LTP) and long-term depression (LTD) [100],[101]. Although synaptic zinc likely plays diverse, important roles in synaptic biology, until recently, few investigators specifically examined synaptic zinc in ASD patients or animal models.

Several years ago, we found that concentrations of extracellular zinc in the micromolar range, which is attainable during intense neuronal activity, activated metalloproteases in cultured cortical neurons [102]. Such exposure to zinc resulted in a concurrent increase in the release of proBDNF and its conversion to mature BDNF, both of which were metalloprotease dependent. These results raised the possibility that synaptically released zinc might activate BDNF/TrkB signaling in a metalloprotease-dependent manner. Interestingly, one study demonstrated that zinc can also transactivate TrkB in BDNF-independent manner [103]. Since metalloprotease activation and BDNF upregulation around synapses may contribute to the development of ASD, the possible role of synaptic zinc in the pathogenesis of ASD may need to be tested.

The first clue that synaptic zinc might play a role in ASD was provided by the observed association of mutations in Shank genes (Shank 1–3) with ASD [11],[104],[105]. Members of the Shank family are postsynaptic scaffold proteins that may play a key role in synapse stability and plasticity through formation of polymers [106]. A recent report demonstrated that zinc rapidly induces Shank3 polymerization [107], leading to the suggestion that synaptic zinc is a key regulator of Shank protein polymerization. A possible role for Shank proteins in normal brain development is suggested by the observation that haploinsufficiency of Shank3 causes Phelan-McDermid syndrome, which is characterized by mental retardation and autistic behavior [108],[109]. Since a Shank3 deficiency may cause ASD, it is reasonable to infer that a synaptic zinc deficiency, which may cause a defect in Shank3 polymerization, might also contribute to synaptic dysfunction in ASD. In fact, a deficiency of dietary zinc was reported to recapitulate several features of autism in mice, perhaps by dysregulating activity-dependent changes in Shank2 and Shank3 levels [104]. These findings suggest the possibility that downregulation of synaptic zinc might also contribute to ASD pathogenesis. However, at least in animal studies, a zinc deficiency results in microcephaly [110]. Hence, although a deficiency in synaptic zinc may cause an autistic phenotype, is not readily compatible with the increased brain size or hyperconnectivity observed in ASD. Accordingly, we considered the possibility that knockout of Shank genes enhances, rather than decreases, ZnT3 expression through a negative feedback, compensatory mechanism. Consistent with this supposition, our preliminary result suggested that at 3 weeks of age levels of free zinc and ZnT3 protein in Shank2-null brains may be higher than in controls (unpublished). Synapses that are deficient in Shank proteins may attempt to restore the function of the Zinc-Shank pathway by increasing presynaptic zinc levels; the undesirable consequence of this compensatory response may be excessive metalloprotease activation and upregulation of BDNF at the synapse. Hence, although the synaptic dysfunction remains, the number of neurons and synapses may be upregulated, resulting in megalencephaly and hyperconnectivity.

Similar mechanisms may be applicable to other models. As discussed above, thimerosal and valproate are capable of causing zinc dyshomeostasis. It would thus be interesting to determine whether zinc dyshomeostasis also occurs in other models of ASD, especially those that are linked to megalencephaly and hyperconnectivity.

#### The ZMB axis hypothesis

On the basis of published findings, described above, and our own preliminary results, we propose the novel hypothesis that an abnormality in the putative zinc-metalloprotease-BDNF (ZMB) axis plays a crucial role in the development of ASD. As discussed above, this hypothesis is easily testable in animal models and, if validated, could mechanistically account for megalencephaly and hyperconnectivity—puzzling aspects of ASD that are currently unresolved. To date, evidence for this hypothesis is admittedly sparse. Still, considering the immensity of the problems posed by ASD in current neuroscience and medicine, we believe the hypothesis is worth pursuing.

**Figure 2:**
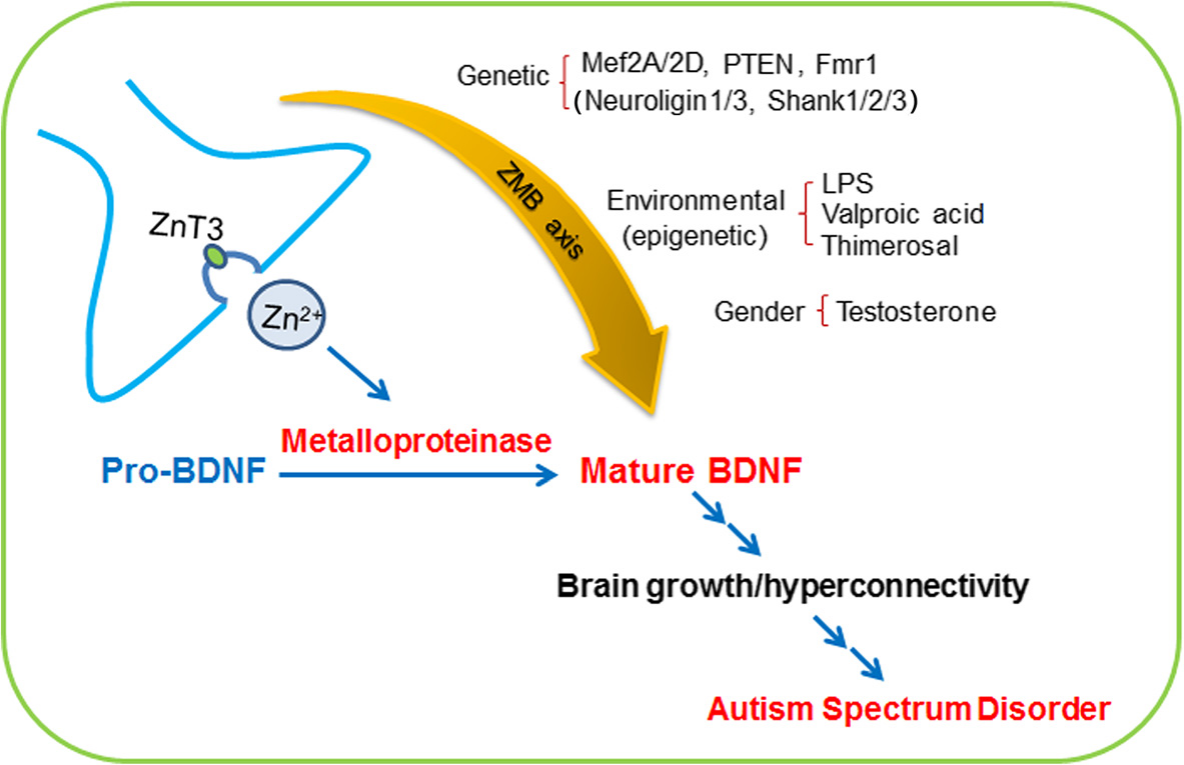
**The ZMB axis model of ASD.** Synaptic zinc may be developmentally modulated by certain genetic, epigenetic, or gender risk factors for ASD. Consequent aberrant activation of metalloproteases may result in prolonged upregulation of BDNF. The resulting enhancement in neurotrophic influences may underlie brain overgrowth and hyperconnectivity, which may contribute to ASD.

The central feature of the hypothesis is that diverse causes of ASD—environmental or genetic—converge on upregulation of the ZMB axis during early brain development. Whereas upregulation of BDNF may result directly from epigenetic changes, as are possibly induced by the known histone deacetylase inhibitor valproate, in many cases, perhaps even in the valproate model, metalloprotease activation may be involved. Inflammation may release synaptic zinc and activate metalloproteases in microglial cells. Environmental toxins such as thimerosal may also release synaptic zinc and, at the same time, directly activate neuronal and/or microglial metalloproteases (Figure 2). Activation of metalloproteases may cause long-lasting activation of BDNF/TrkB signaling, which plays a central role in megalencephaly and hyperconnectivity in young ASD patients, at least in part through epigenetic changes.

### Testing the hypothesis

Tests of the ZMB hypothesis should begin with various animal models of ASD. Three parameters—synaptic zinc, MMP activity and BDNF expression levels—can be relatively easily measured. First, it should be determined whether synaptic zinc or ZnT3 affects outcome in these ASD models. For instance, ZnT3 dosage effects could be tested by crossing ZnT3^+/−^ male and female mice to produce ZnT3^+/+^, ZnT3^+/−^ and ZnT3^−/−^ offspring. Accordingly, injection of valproate into a pregnant mouse should reveal whether the levels of ZnT3 or synaptic zinc affect manifestation of the ASD phenotype. MMP activity and BDNF levels could be measured concurrently by zymography and Western blotting/immunocytochemistry, respectively. If the hypothesis is correct, ZnT3-null offspring should exhibit diminished MMP activation and lower BDNF expression than ZnT3 WT or heterozygous mice.

Shanks are postsynaptic proteins whose expression may be controlled by zinc levels [104],[107],[111]. However, it has not been shown whether synaptic zinc is reciprocally modulated by Shank proteins. One intriguing possibility would be the existence of bilateral crosstalk between Shank proteins and synaptic zinc. This possibility could be tested by examining Shank-null mice for altered ZnT3 or synaptic zinc.

### Implications of validating the ZMB axis hypothesis

If validated, this theory could be translated to human cases, facilitating the search for early biomarkers as well as therapeutic measures capable of halting progression of the ASD pathology.

It has been demonstrated that levels of BDNF in the CNS and peripheral blood (platelets) are closely correlated [43],[67],[112]. However, blood BDNF level alone may not provide a sufficiently specific test, since other causes of mental retardation also can increase BDNF levels in blood [113]. If excess synaptic zinc release and MMP activation occur in early ASD brains, these changes may be detectable in CSF or even in blood. Accordingly, the profiles of all three parameters could be used to supplement blood or CSF tests, strengthening diagnostic specificity. Of course, appropriate translational studies will ultimately be required to validate the usefulness of this approach as a diagnostic tool.

If early biochemical diagnosis is indeed feasible, then preventative measures may be developed. Provided that the ZMB axis hypothesis is validated, drugs that interfere with actions of synaptic zinc such as clioquinol, or metalloprotease inhibitors such as minocycline, may prove effective in reducing the incidence of ASD. These approaches could, and should, be tested in various animal models of ASD. Successful results may be readily translatable to humans, since some of these drugs are currently in clinical use.

In addition to its practical significance, validating the ZMB axis hypothesis would likely provide insight into normal brain development and open up new avenues for the study of ASD mechanisms.

## Conclusion

It has long been known that some ASD children have large heads/brains, but the clinical and/or neurobiological significance of this finding has received relatively little scrutiny until recently. Pathological studies have shown that both gray and white matter increase in volume, suggesting that ASD brains may be under increased trophic influence. This finding is made more interesting by the recent demonstration that ASD brains are likely hyperconnected, which further supports the concept of increased trophic influence. An increasing body of evidence suggests that BDNF upregulation plays a key role in the increased trophic effects in ASD. In several animal models of ASD, BDNF expression is significantly increased. Moreover, in human ASD patients, blood levels of BDNF may be increased. Case control studies on hair samples from young ASD patients also described increased level of zinc [114],[115]. Although all these changes are likely interconnected, how these changes are brought about in ASD remains largely unclear. In this paper, we have proposed the novel hypothesis that abnormalities involving the ZMB axis may play a pivotal role in the hyperconnectivity and megalencephaly observed in ASD. Validating this hypothesis may shed light on the pathogenic mechanisms of ASD and aid in the identification of biomarkers and the development of preventive/therapeutic strategies.

## Abbreviations

ASD: Autism spectrum disorder

BDNF: Brain-derived neurotrophic factor

ZMB: Zinc-metalloprotease-BDNF

fMRI: Functional magnetic resonance imaging

## Competing interests

The authors declare that they have no competing interests.

## Authors’ contribution

JYK conceived and organized the central idea and wrote the manuscript. JSL, HRB, and MHY performed experiments, gathered data, and wrote the manuscript. All authors read and approved the final manuscript.
